# Kinase inhibit region of SOCS3 attenuates IL6‐induced proliferation and astrocytic differentiation of neural stem cells via cross talk between signaling pathways

**DOI:** 10.1111/cns.13992

**Published:** 2022-10-10

**Authors:** Jing An, Ruo‐Lan Tan, Xiao‐Xuan Hu, Zhen‐Lu Cai, Mei‐Qi Sun, Qian Ge, Wen Ma, Hui‐Liang Li, Hai‐Xia Lu

**Affiliations:** ^1^ Department of Neurobiology, School of Basic Medical Sciences Xi'an Jiaotong University Health Science Center Xi'an China; ^2^ Faculty of Medical Sciences, Wolfson Institute for Biomedical Research University College London London UK

**Keywords:** astrogliosis, brain injury, kinase inhibit region of SOCS3, neural stem cell, signaling pathway

## Abstract

**Aims:**

Efficiency of neural stem cells (NSCs) therapy for brain injury is restricted by astrogliosis around the damaged region, in which JAK2/STAT3 signaling plays a key role. The SOCS3 that can directly inhibit JAK/STAT3 pathway. Here, we investigated the effects of a fusion peptide that combined kinase inhibitory region (KIR) of SOCS3 and virus trans‐activator of transcription (TAT) on biological behavior of cultured NSCs under inflammatory conditions.

**Methods:**

NSCs were isolated from embryonic brain of SD rats, TAT‐KIR was synthesized, and penetration rate was evaluated by flow cytometry (FACS). CCK8, immunostaining, and FACS were used to detected of TAT‐KIR on the proliferation of NSCs. The expressions of GFAP and β tubulin III positive cells induced by IL6 with/without TAT‐KIR were examined by immunostaining and Western blotting to observe the NSCs differentiation, and the effect of TAT‐KIR on signaling cross talk was observed by Western blotting.

**Results:**

Penetration rate of TAT‐KIR into primary cultured NSCs was up to 94%. TAT‐KIR did not affect the growth and viability of NSCs. It significantly reduced the NSCs proliferation that enhanced by IL‐6 stimulation via blocking the cell cycle progression from the G0/G1 to S phase. In addition, TAT‐KIR attenuated astrocytic differentiation and kept high level of neuronal differentiation derived from IL‐6‐induced NSCs. The fate of NSCs differentiation under inflammatory conditions was affected by TAT‐KIR, which was associated with synchronous inhibition of STAT3 and AKT, while promoting JNK expression.

**Conclusion:**

TAT‐KIR mimetic of SOCS3 could be a promising approach for brain repair via regulating the biological behaviors of exogenous NSCs.

## Graphical abstract

Differentiation fate of NSCs is regulated by TAT‐KIR. TAT‐KIR inhibits the upregulation of p‐AKT and p‐STAT3 synchronously after IL‐6 induction, giving rise to reduced differentiation of NSCs toward astrocytes. The level of p‐JNK is elevated by IL‐6 or/and TAT‐KIR respectively via different ways, which probably coincided with the increased neuronal differentiation of NSCs. TAT‐KIR could keep the level of p‐ERK at a balance between normal and inflammatory states.

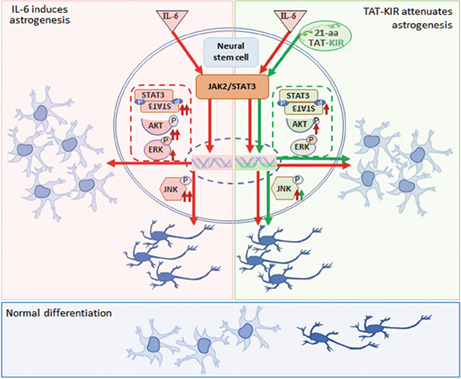

## 1 INTRODUCTION

Brain injury is a common disease with poor prognosis. The outcomes of brain injury result from the degree of destruction or degeneration of neuronal plasticity.^1^, ^2^ NSCs are capable of self‐renewal and differentiating into diverse types of neural cells. It not only provides a cellular reservoir for the replacement of lost/damaged cells, but also possesses several intrinsic capacities to release some neurotrophic factors.^3^, ^4^ Therefore, NSCs transplantation has been considered as an ideal therapeutic strategy for brain injury. According to the characteristics of NSCs, during neural development, NSCs differentiate into specific types of neural cells in response to the local developmental cues.^5^ Along with the maturation, the endogenous NSCs get fewer and quiescent. Up to adulthood, the amount of NSCs is limited and insufficient to compensate for the cell loss after injury.^6^ Therefore, exogenous NSCs are required and considered as a key role for cell replacement after brain injury.^2^, ^7^ Although some studies have presented that neuronal functions could be improved after NSCs implantation, NSC‐based cell therapy for brain injury still faces multiple challenges.^2^, ^7^, ^8^, ^9^ One of the challenges is that the local inflammatory environment induces astrogliosis and hinders the neuronal regeneration and limits the therapeutic effect of NSCs transplantation.^10^ Therefore, effective alleviation of local inflammatory astrogliosis and promotion of neuronal regeneration could be more stirring for neuronal function restoration after brain injury.^11^, ^12^

Janus kinase 2/signal transducer and activator of transcription 3 (JAK2/STAT3) pathway is implicated in a variety of inflammatory response under multiple physiological and pathological conditions. JAK2/STAT3 is highly expressed during development and plays important roles in embryonic cell growth.^13^, ^14^ In adulthood, JAK2/STAT3 pathway is also responsible for proliferation and differentiation of exogenous neural cells in vivo and in vitro treating for most neural disorders, including epilepsy, brain cancer, lesion, ischemia, and neurodegenerative disease.^10^, ^11^, ^12^ It is involved in both neurogenesis and neuroregeneration to regulate NSCs' biological behaviors.^15^, ^16^, ^17^, ^18^ Activation of JAK2/STAT3 pathway by inflammatory cytokines, such as interleukin 6 (IL‐6), leukemia inhibit factor (LIF) and interferons (IFNs),^10^, ^19^ triggers the differentiation of NSCs into glial cells, particularly astrocytes. Conversely, repressing JAK2/STAT3 could reduce astrogliosis and enhance neuronal differentiation.^18^, ^20^, ^21^, ^22^

The one of most useful elements for regulation JAK2/STAT3 pathway is inevitably involved in suppressor of cell signaling 3 (SOCS3).^23^, ^24^, ^25^ SOCS3 is a 225 amino acid protein, and it is known to suppress JAK2 activity through direct binding to the JAK2 catalytic center and promotion of the proteasome degradation of JAK2.^25^ It directly inhibits IL‐6‐induced activation of JAK2/STAT3 inflammatory pathway via SOCS3/JAK2/gp130 complexes.^23^, ^26^ Therefore, regulation of SOCS3 is considered as a candidate approach to reduce brain astrogliosis and promote neuron survival both in vitro and in vivo.^21^, ^22^, ^27^, ^28^, ^29^ As well known, the kinase inhibitory region (KIR) is the central domain of SOCS3 and consists of 8–12 amino acid sequences that directly inhibit JAK2.^23^ Currently, there are no studies on direct use of this small peptide fragment KIR to inhibit the differentiation of NSCs into glial cells in exogenous NSCs niche.

In current study, we directly applied KIR of SOCS3 to inhibit the activation of JAK2/STAT3 signaling and then subsequently to observe the biological behaviors of NSCs in inflammatory condition. A fusion peptide was constructed by using TAT to lead the penetration of cell membrane,^30^ and the IL‐6 was used to induce the inflammatory responses. We found that peptide TAT‐KIR as the mimetic of SOCS3 has the capacity to enter NSCs and help to inhibit the excessive proliferation of NSCs in inflammatory condition. TAT‐KIR attenuates astrocytic differentiation of NSCs by inhibiting STAT3 and AKT in sync, while promoting neuronal differentiation via upregulation of JNK2 in inflammatory condition. This study tested the feasibility of TAT‐KIR applied to NSCs' replacement therapy in vitro to provide a potential strategy for repair of brain injury.

## 2 MATERIALS AND METHODS

### 2.1 Animals

Pregnant Sprague‐Dawley (SD) rats were maintained under a standard 12 h dark–light cycle in a controlled temperature (22 ± 1°C) with free access to food and drink. All procedures involving animal work conformed to the ethical guidelines of the NIH Regulations for Experimentation on Laboratory Animals and monitored by the Institutional Animal Care and Use Committee (IACUC) of Xi'an Jiaotong University under protocol number 2021‐280.

### 2.2 Embryonic NSCs isolation and culture

Tissues were dissected from the cerebral cortex of SD rat embryos on embryonic day 14 (E14). Single‐cell suspension was acquired by gently trituration with pipette. Cells were cultured in DMEM/F12 (the Dulbecco's modified Eagle's medium and Ham's F12, 1:1) serum‐free growth medium that contained 10 ng/ml of the basic fibroblast growth factor, 20 ng/ml of the epidermal growth factor, 1% penicillin, 1% streptomycin, 1% N2, 2% B27 supplement (all from Invitrogen), and incubated in 5% CO_2_ at 37°C, following the standard protocol^31^ and optimized in our laboratory.^32^, ^33^ Cells were sub‐cultured after 5–7 days in vitro (DIV) when the spheres were clearly visible.

### 2.3 NSCs identification

For observation of spontaneous differentiation, the cell aggregates were trypsinized into single cells and seeded on poly‐L‐lysine‐coated coverslips at a density of 0.5 × 10^4^ cells/well in 24‐well plates. Then those cells were cultured in differentiation medium contained DMEM/F12 with 1% fetal bovine serum (Gibco), 1% N2 and 2% B27 supplement for 7 days. Neurospheres and differentiated cells were fixed with 4% paraformaldehyde (PFA) in a 0.1 M sodium phosphate buffer solution (PBS, pH 7.4) for 30 min followed by immunocytochemical staining. The cells were permeabilized in 0.3% Triton X‐100 (Sigma‐Aldrich) for 20 min and incubated with 10% normal goat serum for 1 h at room temperature. After blocking, cells were then incubated with different types of primary antibody, including mouse anti‐nestin (#MAB353, Millipore, 1:200), rabbit anti‐glial fibrillary acidic protein (GFAP) (#ab7260, Abcam, 1:500), mouse anti‐β tubulin III (#MAB1637, Millipore, 1:200), and mouse anti‐oligodendrocyte 4 (#MAB345, Millipore, 1:50) at 4°C overnight. On the following day, after thoroughly washing with PBS, the cells were then incubated with Alexa Fluor 594‐conjugated goat anti‐rabbit IgG (#SA00006‐4, Proteintech, 1:500), Alexa Fluor 488‐conjugated goat anti‐rabbit IgG (#SA00006‐2, Proteintech, 1:500), or Alexa Fluor 488‐conjugated goat anti‐mouse IgG (#SA00006‐1, Proteintech, 1:500) in PBS for 2 h and stained with DAPI (#H‐1200, Vector Laboratories, 0.1 μg/ml) for 20 min at room temperature. Slides were observed with fluorescence microscope Olympus BX‐51.

### 2.4 Fusion peptide TAT‐KIR synthesizing

TAT protein transduction domain was used as a cargo to deliver KIR of SOCS3 into NSCs. The fusion peptide TAT‐KIR was synthesized (Shanghai Chutai biotechnology) and labeled with Fluorescein Isothiocyanate (FITC). The sequence of the fusion peptide (TAT‐KIR) was RKKRRQRRR‐LR‐LKTFSSKSEYQL‐V (RKKRRQRRR was for TAT and LKTFSSKSEYQL was for KIR). A scrambled fusion peptide (TAT‐scamble) RKKRRQRRR‐LR‐LSTFESKESLQE‐V was also synthesized and set as control (Figure 2A). The stored concentration of fusion peptide was 60 μM (−20°C, protected from light), and the working concentration was 3 μM.

### 2.5 Penetrating rate of TAT‐KIR into NSCs analysis

The penetration rate of the fusion peptides was measured via FACS analysis. NSCs 2 × 10^5^ cells/well were cultured in 6‐well plate with growth medium for 3 days. NSCs were randomly allocated in the experimental groups: TAT‐KIR‐FITC and KIR‐FITC. Fusion peptides were added into the NSCs (3 μM) and incubated for 30 min in dark. Then NSCs were washed with PBS to remove the non‐penetrated peptides before the measuring of FITC fluorescence by FACS.

### 2.6 NSCs survival and proliferation analyses

NSCs survival and growth were analyzed by using cell counting kit‐8 (#CK04, Dojindo, CCK‐8). NSCs were randomly allocated in the Control, TAT‐scramble, TAT‐KIR, IL‐6, and TAT‐KIR+IL6 groups. All NSCs seeded into 96‐well plates at 5 × 10^4^ cells/ml and cultured in growth medium for 24 h. IL‐6 in the concentration of 100 ng/ml was applied to mimic inflammatory condition. Thirty minutes later, TAT‐KIR or TAT‐scramble was added into the culture system. Cell viability of each group was observed by Universal Microplate Spectrophotometer (QuantTM, BioTek) at first, third, and fifth day after peptide administration (Figure 3A). Optical density (OD) values at 450 nm were measured. Immunocytochemistry staining with ki67 (#ab16667, Abcam, 1:300) was used to assess NSC proliferation. Cell cycles were analyzed by FACS at third day after peptide administration. The rates of cells in G0/G1, S, and G2/M were measured, and the proliferation index (PI) {PI = (S + G2/M)/(G1/G1 + S + G2/M) × 100%} was calculated, respectively. In each independent experiment, the procedures were carried out in triplicate.

### 2.7 NSCs differentiation assessment

NSCs at 1 × 10^5^ cells/ml were seeded into 24‐well plates allocated randomly in Control, TAT‐KIR, IL‐6, and TAT‐KIR+IL6 groups. After 24 h culture with differentiation media, IL‐6 and TAT‐KIR were added into the medium as above order and reapplied every other day (Figure 4A). Cells were harvested at 7 DIV. Immunocytochemical staining was performed with different antibodies, including GFAP and β Tublin III. Stained cells were observed with fluorescence microscope (BX‐51, Olympus) at an operating temperature below 25°C. Then the numbers of GFAP positive and β Tubulin III positive cells were counted, and the percentages of differentiated cells were calculated by using Image J (version 1.61).

### 2.8 Western blotting analysis

NSCs at 1 × 10^6^ cells/ml in 6‐well plate were treated with IL‐6 and TAT‐KIR as above. Cells were harvested at 3 h, 6 h, 12 h, 24 h, or 7 days after treatment. Cells were suspended in lysis buffer containing 150 μl RIPA (#89900, Pierce) protease and phosphatase inhibitor cocktail (#4693159001 and 04906845001, Roche). Cell lysates were incubated on ice for 20 min and centrifuged at 12,000 *rpm* for 15 min at 4°C. The proteins in supernatant were separated by electrophoresis with 10%–12% sodium dodecyl sulfate polyacrylamide gel (SDS‐PAGE) and then transferred onto 0.22 μm polyvinylidene difluoride membranes (PVDF; #IPVH00010, Millipore). The membranes were blocked in 5% BSA dissolved in Tris‐buffered saline containing 0.1% Tween‐20 for 2 h at room temperature and then were incubated overnight at 4°C with primary antibodies, including GFAP (#ab7260, Abcam, 1:1000), β Tublin III (#MAB1637, Millipore, 1:1000), and STAT3, p‐STAT3, P38, p‐P38, ERK1/2, p‐ERK1/2, AKT, p‐AKT, JNK2, p‐JNK2(#4904, 9145, 8690, 4511, 4695, 9101, 4685, 4060, 9258, 4668, Cell Signaling, all in 1:1000) and β‐actin (#A01263, Boster Biological Technology, 1:20,000). After washing, the membranes were incubated with the secondary antibodies (HRP conjugated anti‐rabbit or anti‐mouse IgG) for 2 h at room temperature. The membranes were visualized by an enhanced chemiluminescence (ECL) kit. All data were analyzed with Image J (version 1.61).

### 2.9 Statistical analysis

All experiments were performed in triplicate. All data were shown as mean ± standard deviation and analyzed with SPSS 18.0 software. All statistical analyses were performed using Student's unpaired *t* test and one‐way analysis of variance (ANOVA). If the data were homogeneous variance, Least Significant Difference test was used for multiple comparisons of ANOVA; if data were non‐homogeneous variance, Dunnett T3 was performed. Nonparametric Kruskal–Wallis tests were performed with data that were not normally distributed. *p* < 0.05 was considered as statistically significant. Origin (version 11.0) was used for bar graph plotting.

## 3 RESULTS

### 3.1 Fusion peptide TAT‐KIR penetrates into NSCs efficiently

**FIGURE 1:**
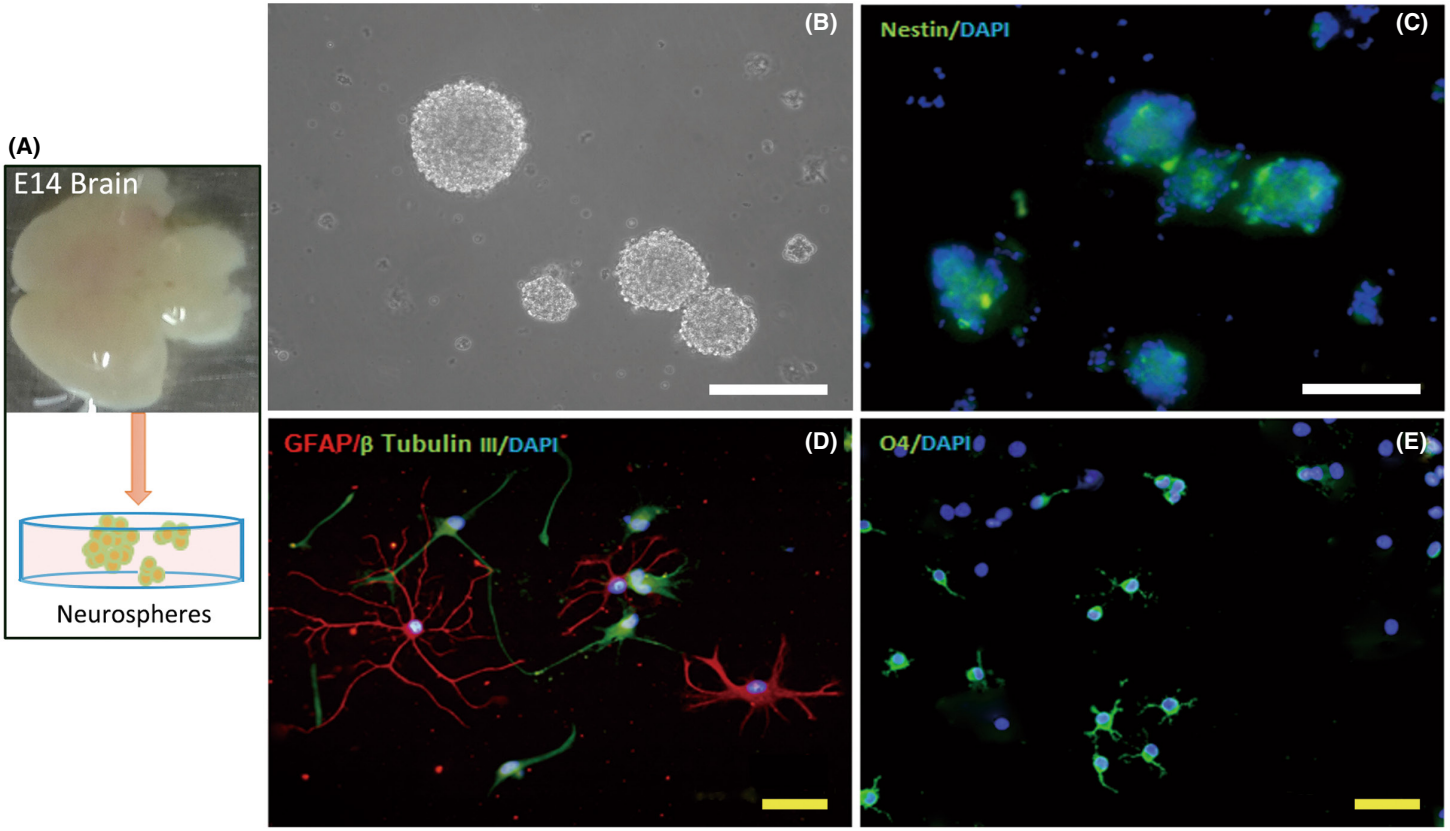
Culturing and identification of rat NSCs. (A) Experimental schematic for collection and cultivation of NSCs. (B) Neurospheres in different sizes were observed in the culture system at 5 DIV. (C) Most of the cells within neurospheres were nestin positive NSCs (green). (D, E) NSCs differentiated into GFAP (red, D), β tubulin III (green, D) and O4 (green, E) positive cells were astrocytes, neurons, and oligodendrocytes respectively. DAPI was used for cell nuclei staining and showed in blue. White scale bar = 200 μm, Yellow scale bar = 50 μm.

As a key role for cell replacement after brain injury, a sufficient number of NSCs are required.^2^, ^7^ According to Fred Gage's research studies and our previous study, NSCs were isolated from developing cerebral cortex of rat embryos on embryonic day 14.5 (Figure 1A) and cultured in DMEM/F12 (1:1) growth medium.^31^, ^33^ Cell aggregates (also called neurospheres) in different sizes developed over a period of 5 days (Figure 1B). The majority of cells in neurospheres were nestin positive NSCs (Figure 1C). Those cells could differentiate into β‐tubulin III‐positive neurons, GFAP‐positive astrocytes, and O4‐positive oligodendrocytes, respectively, after 7 days culture in the differentiation medium (Figure 1D,E). These results confirmed that the cells we cultured are NSCs.

**FIGURE 2:**
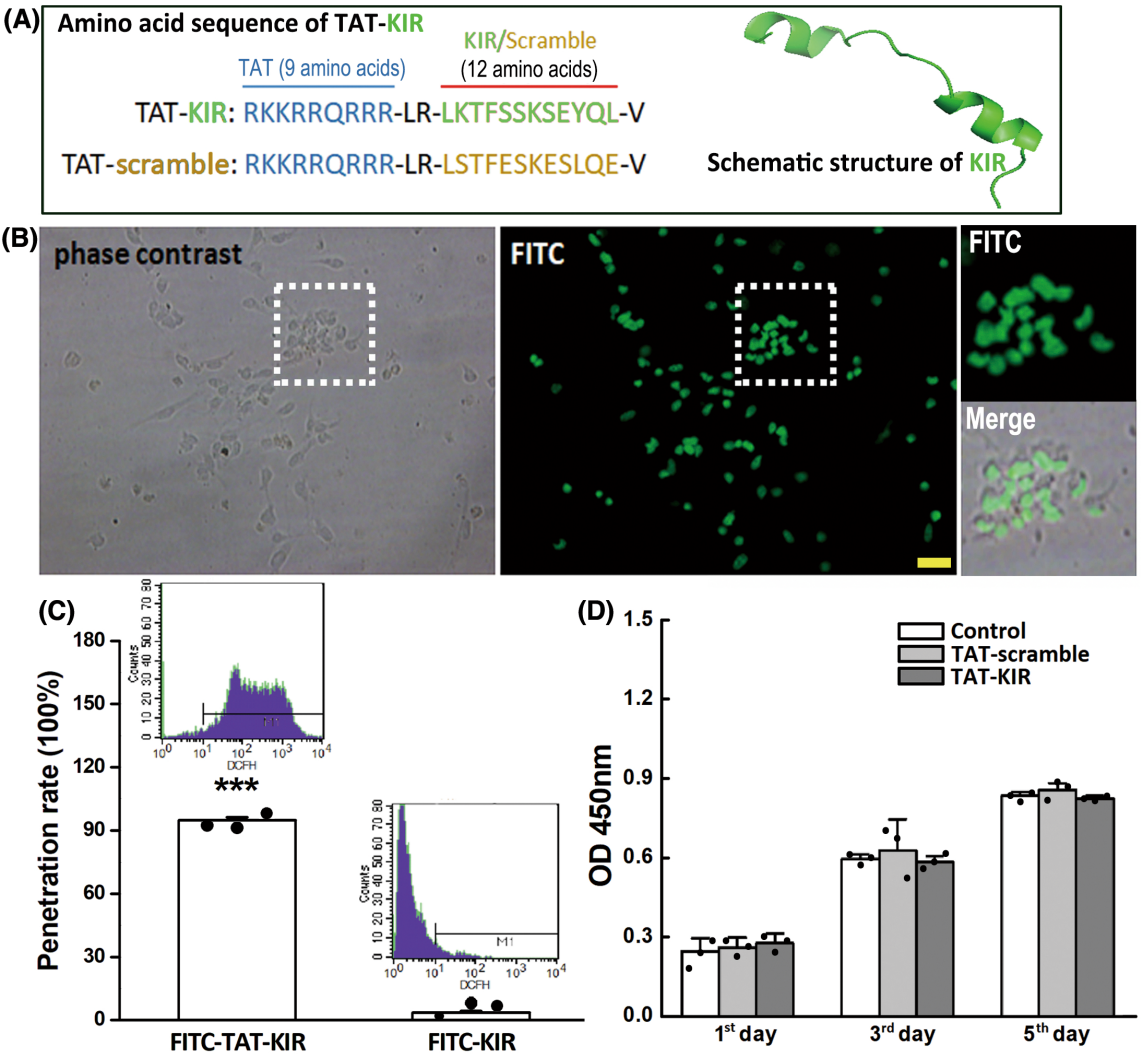
TAT‐KIR penetrates into NSCs efficiently. (A) Sequence and the schematic structure of the fusion peptide TAT‐KIR. (B) TAT‐KIR labeled with FITC in N‐terminal (green) was observed inside of cultured NSCs (Scale bar = 50 μm). (C) The result of flow cytometry showed the penetration rate of TAT‐KIR‐FITC was approximately 94%. It was significantly higher than the rate of KIR‐FITC by nonparametric test (*p* = 0.000). Data were expressed as Mean ± SD (*n* = 3 independent experiments) and were analyzed by Student's unpaired *t* test. ***p < 0.001 compared to the KIR‐FITC group. (D) NSCs viability was measured by CCK‐8 assay, and no significant effect was observed after TAT‐KIR application. Data were expressed as Mean ± SD and were analyzed by one‐way ANOVA followed by Least Significant Difference's test (*n* = 3).

The inflammatory environment induces astrogliosis and impedes the neuronal differentiation of transplanted NSCs in local regions.^10^ Therefore, effective alleviation of local inflammation plays a vital role in NSC‐based therapy.^11^, ^12^ Since SOCS3 plays a critical role in the inhibition of IL‐6‐induced activation of JAK2/STAT3 inflammatory pathway, and KIR is the central role domain of SOCS3.^23^, ^26^, ^34^, ^35^, ^36^ In the current study, we constructed a fusion peptide KIR (12 amino acid) with TAT (9 amino acid) and labeled with FITC in N‐terminal, following our previous work (published in other language in 2014), which had been provided that TAT‐KIR could penetrate into PC12 tumor cells efficiently. The amino acid sequence of TAT‐KIR was RKKRRQRRR‐LR‐LKTFSSKSEYQL‐V (RKKRRQRRR was for TAT and LKTFSSKSEYQL was for KIR) in Figure 2A. A scrambled fusion peptide RKKRRQRRR‐LR‐LSTFESKESLQE‐V was also synthesized and used as control (Figure 2A). After applying the fusion peptide into NSCs' culture system for 30 min, almost all NSCs were labeled with FTIC (Figure 2B). The penetration rate of TAT‐KIR‐FITC was about 94.73 ± 1.51% as shown by flow cytometry (FACS) analysis. It was significantly higher than penetration rate of KIR‐FITC (3.53 ± 0.25%, *p* = 0.000) (Figure 2C), while the growth and viability of NSCs had not been affected as shown by the CCK‐8 assay (Figure 2D).

### 3.2 TAT‐KIR inhibits the IL‐6‐induced excessive proliferation of NSCs

**FIGURE 3:**
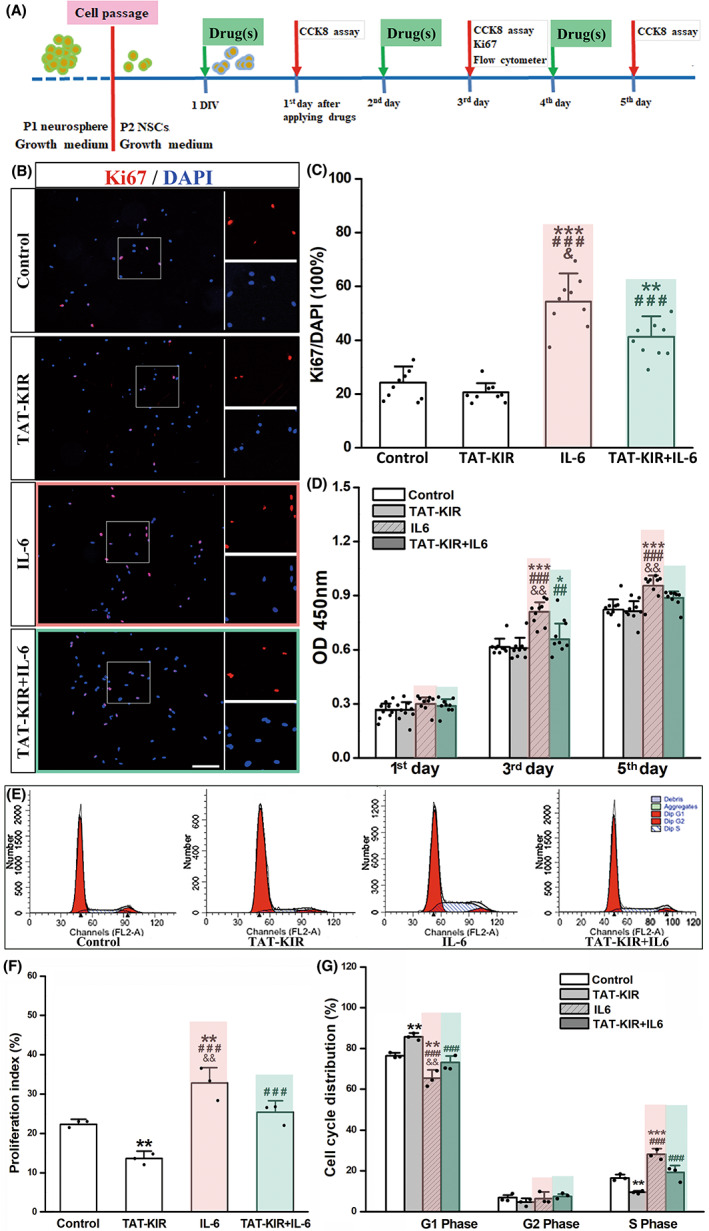
TAT‐KIR abolishes excessive proliferation of NSCs by IL‐6 inducing. (A) Schematic diagram for experimental design. NSCs were cultured in growth medium. (B, C) Ki67 staining showed proliferating NSCs (red) cultured in control, TAT‐KIR, IL‐6, and TAT‐KIR+IL‐6 growth media growing for 3 days (Scale bar = 100 μm, B). Bar graph showed the percentage of Ki67 positive cells (red) to DAPI staining cells (blue) in each group (*n* = 9 figures from three independent experiments, C). The IL‐6‐induced proliferation of NSCs was declined by TAT‐KIR application. The rate of Ki67/DAPI in IL‐6 group (*p* = 0.000) and TAT‐KIR+IL‐6 group (*p* = 0.000) was significantly increased compared with that of control, while the rate of TAT‐KIR+IL‐6 group showed reduction (*p* = 0.047) compared with IL‐6 group. (D) OD450 value of NSCs was measured in different growth media at first, third, and fifth day after drug(s) application. Viability of NSCs was no affected after TAT‐KIR application in normal situation. But TAT‐KIR dragged the level of OD values elevated by IL‐6 induction (*n* = 9, triplicate for three independent samples) at third day (*p* = 0.001) and fifth day (*p* = 0.009) compared with those of IL‐6 group. (E–G) FACS evaluated cell cycle of NSCs cultured in control, TAT‐KIR, IL‐6, and TAT‐KIR+IL‐6 growth media. The percentage of PI in TAT‐KIR+IL‐6 was dramatically reduced vs. IL‐6 group (*n* = 3 independent samples, *p* = 0.007, F). Cell cycle distribution showed the percentage of IL‐6 nduced NSCs in S phase was abolished by TAT‐KIR, while the G0/G1 percentage was upregulated by TATKIR (*n* = 3 independent samples, G). The percentage of S phase showed reduction in TAT‐KIR+IL‐6 vs. IL‐6 group (*p* = 0.001). Data were expressed as Mean ± SD and were analyzed by one‐way ANOVA followed by Least Significant Difference's test. *p ˂ 0.05, **p ˂ 0.01, ***p ˂ 0.001 vs. control group. ^##^ *p* ˂ 0.01, ^###^ *p* ˂ 0.001, vs. TAT‐KIR group. ^&^ *p* < 0.05, ^&&^ *p* < 0.01 vs. IL‐6 + KIR group.

It has been reported that the level of inflammatory cytokines, such as IL‐6, LIF, and IFNs, significantly elevated after brain injury.^10^ In the current study, IL‐6 was used to mimic the inflammatory response after brain injury and to active JAK2/STAT3 pathway^10^, ^19^ (Figure 3A). Consistent with the existed reports that IL‐6 could enhance the proliferation of neural progenitors.^37^ In our study, the primary cultured embryonic NSCs' proliferation was dramatically promoted after IL‐6 induction for 3 days, as shown by the percentage of the Ki67 positive proliferating cell (54.33 ± 10.29% vs. control 24.27 ± 6.06%, *p* = 0.000). No significant difference between normal control (24.27 ± 6.06%) and TAT‐KIR‐treated group (20.67 ± 3.74%). However, the elevation induced by IL‐6 was significantly reduced after TAT‐KIR application (41.33 ± 7.72% vs. IL6 group, *p* = 0.047; Figure 3B,C). Similar results were observed in regarding to NSCs growth as shown by CCK‐8 assay (Figure 3D). IL‐6‐induced OD values of NSCs were strikingly increased from normal level at third and fifth day after drug application (OD value of third day 0.81 ± 0.06, vs. 0.61 ± 0.05, *p* = 0.000; OD value of fifth day 0.94 ± 0.06, vs. 0.82 ± 0.06, *p* = 0.000). Whereas TAT‐KIR performed together with IL‐6, the elevated levels of OD values were dragged down dramatically observed after treatment for 3 days (OD value of TAT‐KIR+IL6 was 0.70 ± 0.10 vs. OD value of IL6 0.81 ± 0.06, *p* = 0.001) and 5 days (OD value of TAT‐KIR+IL was 0.70 ± 0.10 vs. OD value of IL6 0.94 ± 0.06, *p* = 0.009; Figure 3D).

Furthermore, NSC cycle was investigated by FACS analysis, and the proliferation index (PI) was calculated. The PI value of NSCs dramatically ascended after IL‐6 induction from normal level 23.57 ± 1.38% to 34.61 ± 4.12% (*p* = 0.001), and PI value descended after TAT‐KIR treatment from normal level 23.57 ± 1.38% to 14.34 ± 1.98% (*p* = 0.003). When applying IL‐6 together with TAT‐KIR, the enhancement of PI value induced by IL‐6 was significantly reduced to 26.82 ± 3.04% (vs. IL‐6 group, *p* = 0.007; Figure 3E,F), which is similar with the analysis of Ki67 positive cells (Figure 3B,C). The analysis of cell cycle distribution showed that, after IL‐6 induction, significant more cells move to S phase (IL‐6: 28.14 ± 2.79% vs. con: 16.57 ± 1.67%, *p* = 0.000) and fewer cells stagnated in G0/G1 phase (IL‐6: 65.39 ± 4.12% vs. con: 76.43 ± 1.38%, *p* = 0.001). An opposite result was observed in TAT‐KIR‐treated group. Cells percentage in S phase reduced from 16.57 ± 1.67% (control) to 9.56 ± 0.22% (*p* = 0.003) while cells in G0/G1 phase increased from 76.43 ± 1.38% (control) to 85.66 ± 1.98% (*p* = 0.003) (Figure 3G). Consistent with PI value, when applying IL‐6 together with TAT‐KIR, the alteration induced by IL‐6 was significantly reversed. TAT‐KIR increased the percentage of G0/G1 phase (73.18 ± 3.04% vs. IL‐6 group, *p* = 0.007) and reduced the percentage of S phase (19.31 ± 3.32% vs. IL‐6 group, *p* = 0.001). No difference was found regarding cells in G2/M phase in different groups. Taken together, the excessive proliferation of NSCs induced by IL‐6 was significantly abolished by TAT‐KIR as we expected.

### 3.3 TAT‐KIR attenuates astrocytic differentiation and keeps high level of neuronal differentiation derived from IL‐6‐induced NSCs

**FIGURE 4:**
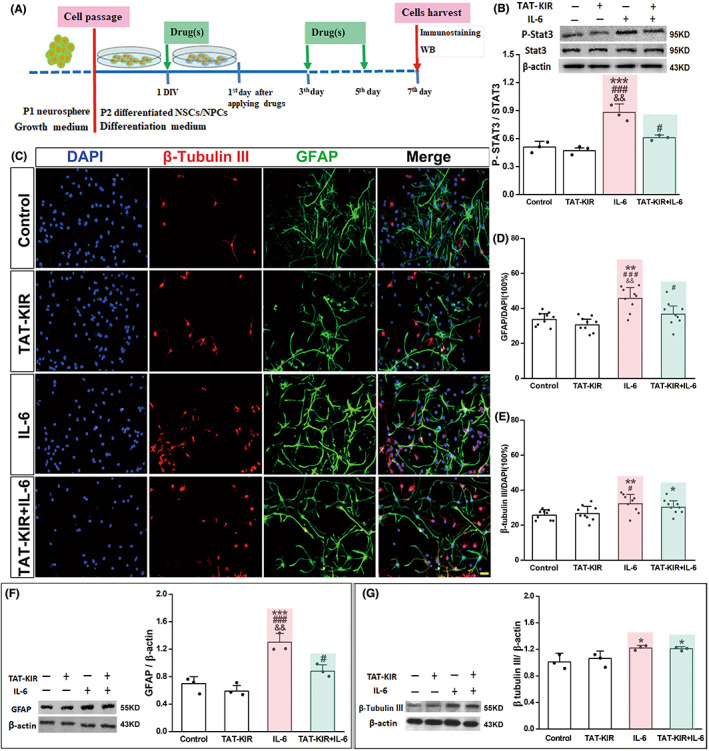
TAT‐KIR attenuates IL‐6‐induced astrocyte activation and promotes neuronal differentiation of NSCs. (A) Schematic diagram of experimental design. NSCs were cultured in differentiation medium for 7 days after treatment. (B) Western blot analyzes the level of the p‐STAT3 protein derived from differentiated NSCs in IL‐6, TAT‐KIR, IL‐6, and TAT‐KIR+IL‐6 groups at 7th day. Rate of p‐STAT3 to STAT3 was decreased when applying TAT‐KIR together with IL‐6 compared with that in IL‐6 group (*p* = 0.001; *n* = 3 independent samples). (C–E) Immunostaining showed that NSCs differentiation (neuron in red and astrocyte in green; Scale bar = 50 μm, C), particularly astrocytic differentiation was enhanced after IL‐6 stimulating, and this effect was significantly reversed by TAT‐KIR treatment (*p* = 0.003, D). Whereas the neuronal differentiation kept a high level in TAT‐KIR treated group vs. Il‐6 group (*n* = 9 for three independent samples, *p* = 0.365, E) (F, G) Western blot analysis showed that GFAP and β tubulin III protein levels were significantly elevated after IL‐6 stimulation. The expression of GFAP reversed back to control lever after TAT‐KIR treatment (*n* = 3, GFAP_TAT‐KIR+IL‐6_ vs. GFAP_con_, *p* = 0.054, F); The expression of β tubulin III protein kept a high level in TAT‐KIR‐treated group vs. control group (*n* = 3, β tubulin III _TAT‐KIR+IL‐6_ vs. β tubulin III_con_, *p* = 0.026, G). Data were expressed as Mean ± SD and were analyzed by one‐way ANOVA followed by Least Significant Difference's test. Scale bar = 25 μm; *p ˂ 0.05, **p ˂ 0.01, ***p ˂ 0.001 vs. control group. ^#^ *p* ˂ 0.05, ^###^ *p* ˂ 0.001 vs. TAT‐KIR group. ^&&^ *p* < 0.01 vs. IL‐6 + KIR group.

IL‐6 also could promote astrogenesis by activating JAK2/STAT3 pathway.^26^ We then explored the possible effects of TAT‐KIR on NSCs differentiation via inhibiting JAK2/STAT3 signaling in normal condition or IL‐6 induced inflammatory environment. As the schematic paradigm showed, NSCs were cultured in differentiation medium for 7 days (Figure 4A). Compared with IL‐6 group, the rate of p‐STAT3 to STAT3 was decreased in TAT‐KIR together with IL‐6 group (Rate_TAT‐KIR+IL‐6_: 0.61 ± 0.025 vs. Rate_IL‐6_: 0.88 ± 0.093, *p* = 0.001) (Figure 4B, Appendix S1 for 4B). The elevated phosphorylation of STAT3 in IL‐6 induced NSCs (Rate_IL‐6_ vs. Rate_con_: 0.51 ± 0.06, *p* = 0.000) was significantly reduced by TAT‐KIR treatment as we expected.

NSCs were cultured in differentiation medium for 7 days. In normal culture condition, TAT‐KIR did not affect NSCs differentiation (Figure 4C–E). Whatever it was neuronal differentiation (Con: 25.78 ± 3.27% vs. TAT‐KIR: 26.78 ± 4.79%, *p* = 0.649) or astrocytic differentiation (Con: 33.78 ± 4.47% vs. TAT‐KIR: 30.67 ± 4.66%, *p* = 0.28), significant difference was not observed. However, in the lL‐6‐induced inflammatory condition, dramatically increased NSCs differentiation was observed, as shown by the percentage of β tubulin III positive (32.33 ± 5.68% vs. con, *p* = 0.005) and GFAP positive cells (45.72 ± 7.50% vs. con, *p* = 0.000). When applying IL‐6 together with TAT‐KIR, such increase of GFAP positive cells was dropped to 36.78 ± 6.81% vs. IL‐6 (*p* = 0.003), and vs. con (*p* = 0.298; Figure 4D). Whereas the percentage of β tubulin III positive cells kept a high level at 30.33 ± 4.39% vs. con (*p* = 0.044), and vs. IL‐6 (*p* = 0.365) (Figure 4E).

The expression of GFAP and β tubulin III at protein level were detected via Western blotting assay to further confirm the effect of TAT‐KIR on NSCs differentiation (Figure 4F,G). When differentiated NSCs treated with TAT‐KIR and IL‐6 at same time, the elevated GFPA expression induced by IL‐6 was significantly reduced (TAT‐KIR+IL‐6: 0.88 ± 0.091 vs. IL‐6: 1.30 ± 0.127, *p* = 0.001; Figure 4F, Appendix S1 for 4F), and the expression of β tubulin III keep on similar state compared with the expression in IL‐6‐treated groups (TAT‐KIR+IL‐6: 1.21 ± 0.025 vs. IL‐6: 1.22 ± 0.044, *p* = 0.859; Figure 4G, Appendix S1 for 4G), which are resemblance to the staining properties of GFAP and β tubulin III proteins in differentiation of NSCs (Figure 4C–E). Together, the excessive astrocytic differentiation of NSCs induced by IL‐6 was significantly attenuated by TAT‐KIR as we expected, while the effect on neuronal differentiation requires further investigation.

### 3.4 Cross talk of different signaling pathways during TAT‐KIR affecting NSCs biological behaviors

**FIGURE 5:**
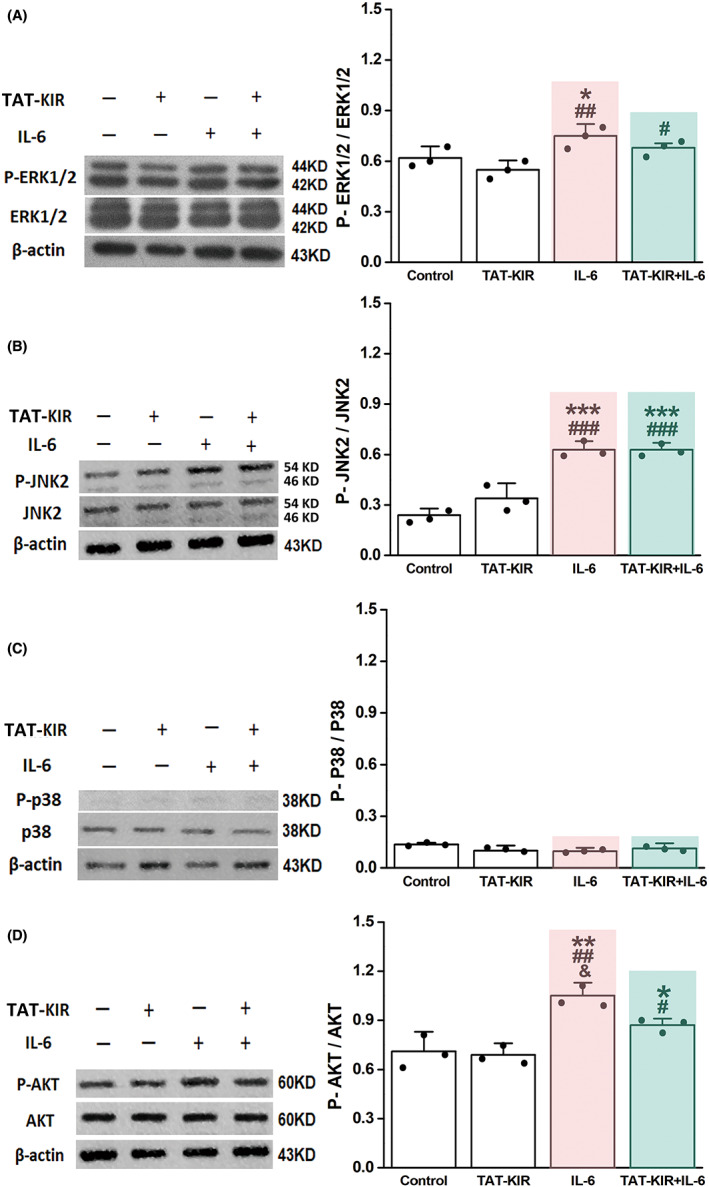
TAT‐KIR regulates phosphorylation levels of MAPK and AKT in inflammatory environment. (A) Western blotting analyzed the quantification of p‐ERK1/2 and ERK1/2 protein expression of NSCs culturing in control, TAT‐KIR, IL‐6, and IL‐6 + TAT‐KIR differentiation media for 7 days respectively. The rate of pERK/ERK remained at high levels after IL‐6 induction with/without TAT‐KIR treatment (*p* = 0.171). (B) The rate of p‐JNK2/JNK2 for IL‐6 induction with/without TAT‐KIR kept similarly high level compared with control group (*p* = 1.000). (C) There were no changes in rate of p‐P38/P38 for the four groups. (D) The rate of pAKT/AKT was elevated after IL‐6 stimulation, and TAT‐KIR treatment reduced the rate significantly (*p* = 0.028). Data were expressed as Mean ± SD and were analyzed by one‐way ANOVA followed by Least Significant Difference's test. *n* = 3 independent samples. *p ˂ 0.05, **p ˂ 0.01, ***p ˂ 0.001 vs. control group. ^#^ *p* ˂ 0.05, ^##^ *p* ˂ 0.01, ^###^ *p* ˂ 0.001 vs. TAT‐KIR group. ^&^ *p* < 0.05 vs. IL‐6 + KIR group.

As the key inhibitory region of SOCS3, KIR can directly inhibit activity of JAK2 and lead to a secondary reduce of STAT3 phosphorylation.^23^ Our results showed that in the normal culture environment, TAT‐KIR did not affect the phosphorylation of STAT3 as well as the astrocytic differentiation of NSCs. Nevertheless, it significantly reduced the IL‐6‐induced excessive elevated p‐STAT3 and the inflammatory reactive proliferation/astrocytic differentiation of NSCs (Figure 4B–E). Since the mitogen‐activated protein kinase (MAPK) signaling pathway is essential in regulating many cellular processes including inflammation, cell stress response, cell division, proliferation, and differentiation.^38^ In order to see the potential cross talk between JAK2/STAT3 and MAPK signaling pathways during TAT‐KIR regulating NSCs biological behaviors, three main members of MAPKs, including extracellular signal‐related kinases ERK1/2, Jun N‐terminal kinases (JNK), and p‐38 proteins were detected. Accompanying with the alteration of NSCs‐differentiation fate and STAT3 phosphorylation (Figure 4B–E) under IL‐6‐induced condition, our results showed that the rate of p‐ERK1/2 to ERK1/2 in NSCs after IL‐6 induction (0.75 ± 0.070) was not significantly different compared with the rate of TAT‐KIR and IL‐6 treatment together (0.68 ± 0.041, *p* = 0.171; Figure 5A, Appendix S1 for 5A). The rate of p‐JNK2 to JNK2 had a different alteration pattern (Figure 5B, Appendix S1 for 5B‐D), the rates kept high level in IL‐6 (0.63 ± 0.049, *p* = 0.000) and TAT‐KIR+IL‐6 groups (0.63 ± 0.042, *p* = 0.000) vs. control group (0.24 ± 0.036). There was not significant difference in rate of p‐JNK2/JNK2 between IL‐6 and TAT‐KIR+IL‐6 groups (*p* = 1.000). In TAT‐KIR group, p‐JNK2 had an increased trend for 7 days differentiation. The responses of p‐JNK2 were more similar to the morphological results of neuronal differentiation of NSCs (Figure 4C,E,G). No change in regarding to p‐38 was found in any of the group (Figure 5C).

Besides MAPKs, PI3K/AKT signaling pathway is also well known for regulation of NSCs proliferation and differentiation under stress.^39^, ^40^ Therefore, the rate of p‐AKT to AKT was detected at the same time, to further explore the cross talk between JAK2/STAT3 and PI3K/AKT. The elevated p‐AKT/AKT of differentiated NSCs induced by IL‐6 (1.05 ± 0.081) was significantly reduced by TAT‐KIR (0.87 ± 0.037, *p* = 0.028) (Figure 5D). Interestingly, the level of p‐AKT/AKT changed almost synchronizes with p‐STAT3/ STAT3 level and the astrocytic differentiation of NSCs in all groups (Figure 4B,F).

**FIGURE 6:**
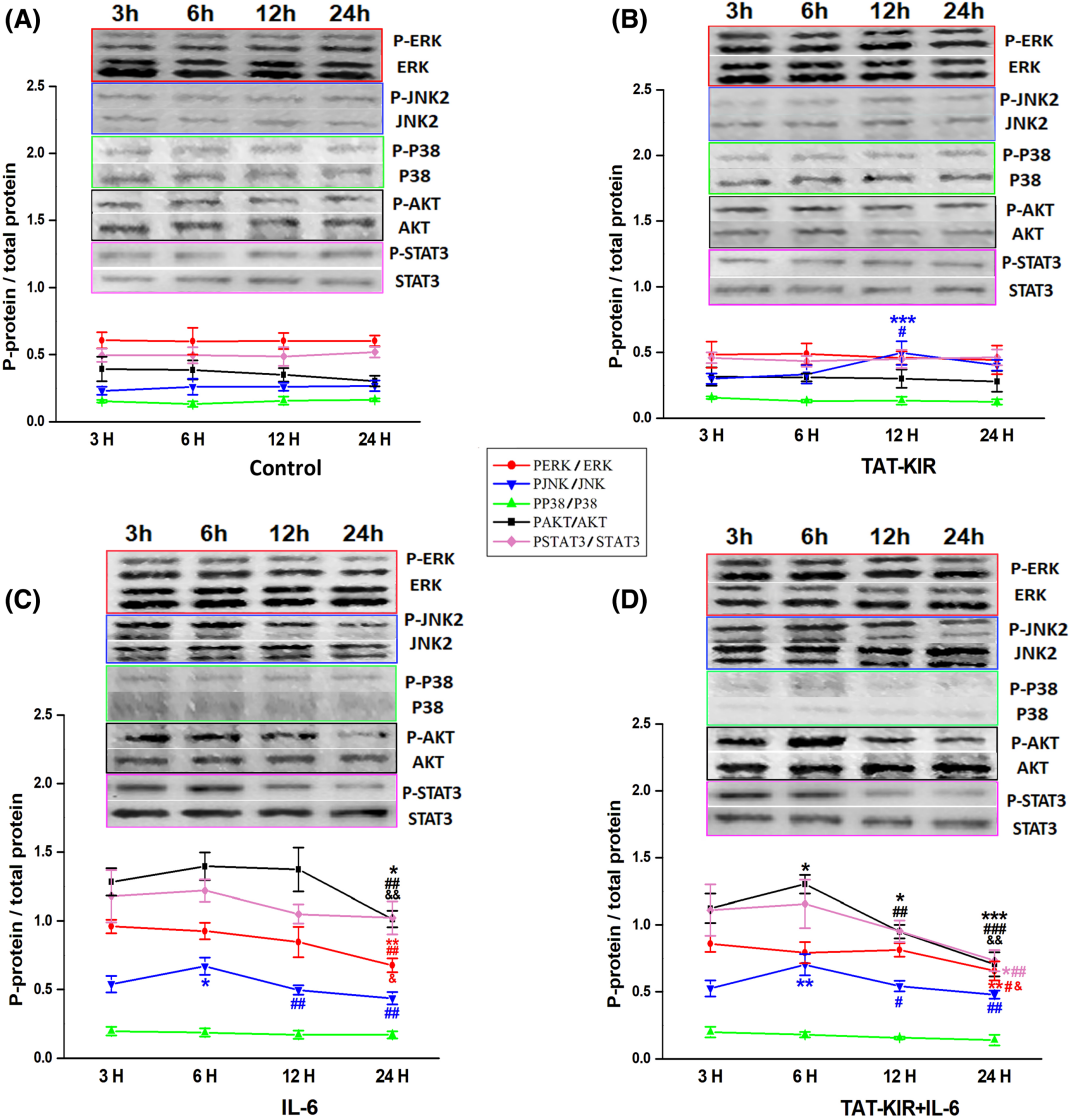
Cross talk of signaling pathways in differentiated NSCs after TAT‐KIR treatment within 24 h. (A) Properties of signaling pathways of differentiated NSCs were cultured in control medium. There were no significant changes between different time points in the normal culture condition. (B) Changes of signaling pathways of differentiated NSCs were cultured in TAT‐KIR medium. Peak level of p‐JNK2/JNK2 in TAT‐KIR group appeared at 12 h. There were no significant changes between different time points in other culture conditions. (C) Regulation of signaling pathways of differentiated NSCs was cultured in IL‐6 medium. The phosphorylation levels of all proteins significantly elevated at 3 h after treatment and maintained at a high level until 12 h, excepting p‐P38/P38. Peak level of p‐JNK2/JNK2 appeared at 6 h, then dropped down quickly. (D) Regulation of signaling pathways of differentiated NSCs was cultured in TAT‐KIR+IL‐6 medium. After 6 h the phosphorylation levels of p‐AKT and p‐STAT3 were decreased by TAT‐KIR in sync. Peak level of p‐JNK2/JNK2 appeared at 6 h, the level returned to 3 h's level after 12 h. The p‐ERK remained at high levels maintained at a high level until 12 h. Red round: rate of p‐ERK/ERK, blue triangle: rate of pJNK2/JNK2, green triangle: rate of p‐P38/P38, black square: rate of p‐AKT/AKT, purple rhombus: rate of pSTAT3/STAT3. 3 h means at 3rd hour point, 6 h means at 6th hour point, 12 h means at 12th hour point, 24 h means at 24th hour point. Data were expressed as Mean ± SD and were analyzed by one‐way ANOVA followed by Least Significant Difference's test. When data did not show normal distribution, Dunnett T3 was used. *n* = 3 independent samples. *p ˂ 0.05, **p ˂ 0.01, ***p ˂ 0.001 vs. 3 h. ^#^ *p* ˂ 0.05, ^##^ *p* ˂ 0.01, ^###^ *p* ˂ 0.001 vs. 6 h. ^&^ *p* < 0.05, ^&&^ *p* < 0.01 vs. 12 h.

To further confirm the cross talk between various signaling pathways and figure out the specific change patterns, the levels of p‐ERK/ERK, p‐JNK/JNK, p‐p38/p38, p‐AKT/AKT together with p‐STAT3/ STAT3 were detected during the first 24 h after TAT‐KIR and/or IL‐6 treatment. Consistent with Figure 5, the level of p38 was incredibly stable in any situation (Figure 6). Regarding the other four proteins, no significant change was found between different time points in the normal culture condition (Figure 6A). However, in the IL‐6‐induced inflammatory environment, the phosphorylation levels of all those four proteins significantly elevated at 3 h after treatment and maintained at a high level until 12 h (Figure 6C). The p‐ERK remained at high levels after IL‐6 induction with/without TAT‐KIR treatment (Figure 6C,D). Interestingly, the peak level of p‐JNK in TAT‐KIR group appeared at 12 h (p‐JNK/JNK_12H_: 0.50 ± 0.090 vs. p‐JNK/JNK_3H_: 0.30 ± 0.040, *p* = 0.005) after treatment while it was advanced to 6 h after treated with IL‐6 (p‐JNK/JNK_6H_: 0.67 ± 0.062 vs. p‐JNK/JNK_3H_: 0.54 ± 0.056, *p* = 0.014; Figure 6B,C). This temporal difference of p‐JNK/JNK in TAT‐KIR+ IL‐6 situation had combined two different patterns from IL‐6 or TAT‐KIR application, respectively (Figure 6D). The elevated p‐AKT and p‐STAT3 expressions of IL‐6‐induced NSCs were dramatically reduced after TAT‐KIR treatment for 3 h (Figure 6C, D). And the synchronous changes of p‐AKT and p‐STAT3 at different time points were also observed in TAT‐KIR‐treated inflammatory environment (Figure 6D, Appendix S1 for 6A‐D).

Together, the cross talk between JAK2/STAT3 and MAPK, PI3K/AKT did exist. In other words, during the process of TAT‐KIR regulating NSCs biological behaviors, ERK1/2 might be involved in the regulation of NSCs proliferation, while JNK and AKT were in the alteration of neuronal and astrocytic differentiation of NSCs, respectively.

## 4 DISCUSSION

SOCS3 plays a key role in inhibition of JAK2/STAT3 via binding to gp130‐related IL‐6 receptors and JAK2 at the same time.^26^ Previous studies have reported that overexpression of SOCS3 by gene editing could prevent reactive astrogliosis and enhance the survival of newborn neurons.^21^, ^22^, ^27^, ^28^, ^29^ KIR as the central role domain of SOCS3 has been proposed to function as a pseudosubstrate of JAKs. It has a potential treatment to regulate the expression of SOCS3 causing JAK/STAT inhibition in bone‐associated inflammatory diseases, influenza, and breast cancer.^34^, ^35^, ^36^ Upon comparison of gene editing approaches, the application of small molecular peptide, such as KIR, is much easier and feasible. However, no study has yet directly used KIR of SOCS3 to regulate neural cellular functions.

In this study, we hypothesized that KIR could be the mimetic of SOCS3 and to inhibit the activation of STAT3 signaling, alter the inflammatory responses, and regulate the biological behaviors of NSCs. A fusion peptide TAT‐KIR (24 amino acids) was constructed and applied to alter the IL‐6‐induced inflammatory effects on NSCs. TAT is an efficient transmembrane peptide widely used for drug delivery.^30^ It could successfully carry various proteins into NSCs and used for neural disorders treatment due to its competitive ability of crossing blood–brain barrier (BBB) and lower cell toxicity.^41^ In the current study, the penetration rate of TAT‐KIR to NSCs was 94%, and no cell toxicity was found. It absolutely met our requirements.

The effects of TAT‐KIR on survival and proliferation of NSCs were explored. Taken the results of cell viability, cell cycle assays, and Ki67 immunostaining together, TAT‐KIR significantly reduced the excessive alteration of NSCs division and proliferation that induced by IL‐6. Notably, more NSCs arrested in G0/G1 phase, and fewer cells moved to S phase after treated with TAT‐KIR. It is consistent with previous reports that stimulation of cytokine receptor enhanced embryonic stem (ES) cell differentiation^14^ and G1 to S cell‐cycle transition.^42^ Activation of STAT3 also exhibited a tendency toward ES cell differentiation.^14^ This may relate to that p‐STAT3 dimer is involved in synthesis of cyclin D1 and mediated the entry of S phase.^14^, ^42^, ^43^ Conversely, inhibition of STAT3 activation resulted in the growth of ES cells as undifferentiated clones.^14^ In current study, our results suggested that KIR helps to keep NSCs in quiescence in normal environment and could reduce the excessive alteration of NSCs in IL‐6‐induced inflammatory environment.

We then sent out to observe the effect of KIR on NSCs differentiation. After induced by IL‐6, astrocytic differentiation of NSCs dramatically increased accompanied with the activation of JAK2/STAT3 signaling pathway. It is consistent with other reports that IL‐6 can activate JAK2/STAT3 signaling pathway and lead to reactive astrogliosis and scar formation after neural injury.^37^ Regulation of reactive astrocytes in inflammatory microenvironment is pivotal for functional performance of NSCs after transplantation. Several methods that regulating IL‐6 and *Stat3* had been used to inhibit astrogliosis. IL‐6‐deficient mice have remarkable reduction of activated astrocytes and increase of late neuronal response after axotomy.^16^ After *Stat3* deletion, NSCs favor to differentiate into neurons rather than astrocytes.^20^ Conditional deletion of *Stat3* mice had a notable lower level astrocytes reaction, which help axons extending in the early stage after contusive spinal cord injury.^21^ Since many reports have used SOCS3 to hinder reactive astrocytes generation,^21^, ^22^, ^27^, ^28^, ^29^, ^44^ in this study, we directly used KIR, the catalytic region of SOCS3, to observe the alteration of NSC biological behaviors in both normal and inflammatory environments. Our data presented that KIR was able to abolish the IL‐6‐induced generation of astrocytes successfully. It demonstrated that KIR played an anti‐inflammatory role via impeding the activation and regeneration of astrocytes. As regards the effect on neuronal differentiation of NSCs, further investigation is required, although the ascent tendency of expression of β tubulin III was observed after TAT‐KIR or IL‐6 treatment.

In addition to JAK2/SATA3, MAPK and PI3K/AKT signaling pathways have also been considered as regulators of proliferation and differentiation of several types of cells.^37^, ^40^ Our results showed that there were cross talks between JAK2/SATA3 and MAPK, PI3K/AKT signaling pathways. ERK is involved in the regulation of both cell proliferation and differentiation.^43^, ^45^ KIR, as the inhibitor of JAK2/SATA3 signals, could reduce the activation of ERK both in normal and inflammatory conditions. While it kept the level of p‐ERK at a balance between normal and inflammatory states. This change was coincided with NSCs proliferation.

JNK and p38 MAPK are the other two members of the MAPKs. They have been implicated in toxicant‐induced apoptosis of neurons.^46^ JNK can keep redox homeostasis in stress signal transduction.^47^ It also is a key regulator for early neuronal differentiation of NSCs via the cross talk with STAT3 pathway in vitro and in vivo.^48^, ^49^ In our study, JNK was activated in a specific way. When the JAK2/SATA3 signal was inhibited by KIR alone, phosphorylation level of JNK elevated at 12 h after treatment. While the JAK2/SATA3 signal was activated by IL‐6, the activation of JNK advanced to 6 h after treatment. Therefore, the effect of TAT‐KIR on upregulating p‐JNK just be hidden under the action of IL‐6. The alteration of p‐JNK coincided with the expression of differentiated neurons derived from NSCs in vitro. P‐38 was considered to mediate cell apoptosis. In the current study, no change was observed in any of the group.

PI3K/AKT pathway has been implicated in proliferation and differentiation of NSCs. Activation of PI3K/AKT could upregulate cell cycle progression mediating G1 to S phase of mitotic cycle in ES cells.^42^, ^43^ In the current study, we demonstrated KIR inhibits the phosphorylation of AKT under inflammatory conditions. This change was coincided with phosphorylation of STAT3 and astrocytic differentiation of NSCs.

One of the main limitations in our study is that we only explored the effects of TAT‐KIR on NSCs behavior in vitro, there is a lack of in vivo evaluation to confirm the in vitro conclusions. The approaches of exogenous stem cell for CNS tissue repair have many challenges.^50^ (1) Drug safety. Although cellular assay of TAT was demonstrated low toxicity,^41^ safety assessment of TAT‐KIR in vivo is necessary. (2) Drug delivery. In vivo experiments, local injection of TAT‐KIR into the injury region of brain would be considered first. However, TAT is competitive in crossing the BBB,^30^ intravenous administration should also be attempted as another attractive method that is easy to use clinically. (3) Additionally, there may be differences between in vitro and in vivo results. Therefore, the therapeutic effect of TAT‐KIR on transplanted NSCs should be evaluated in vivo to provide more support for future clinical trials.

## 5 CONCLUSION

In current work, we evaluated the small molecular fusion peptide TAT‐KIR has the capacity to enter NSCs and helps to keep NSCs in quiescent in non‐inflammatory condition. It significantly impedes the proliferation and astrocytic differentiation of NSCs in IL‐6‐induced inflammatory environment. Meanwhile, TAT‐KIR tends to promote the neuronal differentiation. TAT‐KIR regulates the activation of ERK, JNK, and AKT by inhibiting JAK2/SATA3 signaling pathway, which changes the proliferation and differentiation of NSCs in vitro (Graphical abstract). Our results provided TAT‐KIR could be considered as a promising therapeutic approach for brain repair via regulating the biological behaviors of exogenous NSCs.

## CONFLICT OF INTEREST

The authors declare that they have no conflicts of interest.

## Supporting information


Appendix S1

## Data Availability

The data that support the findings of this study are available from the corresponding author upon reasonable request.
