# Supplementary material for: Kinase inhibit region of SOCS3 attenuates IL6‐induced proliferation and astrocytic differentiation of neural stem cells via cross talk between signaling pathways
Source: CNS Neurosci Ther. 2022 Oct 10;29(1):168–80. doi: 10.1111/cns.13992 (PMC9804055; doi:10.1111/cns.13992)
Supplement: Supplementary file 1 — Appendix S1 [file CNS-29-168-s001.pdf]

Original blot images for Figure 4

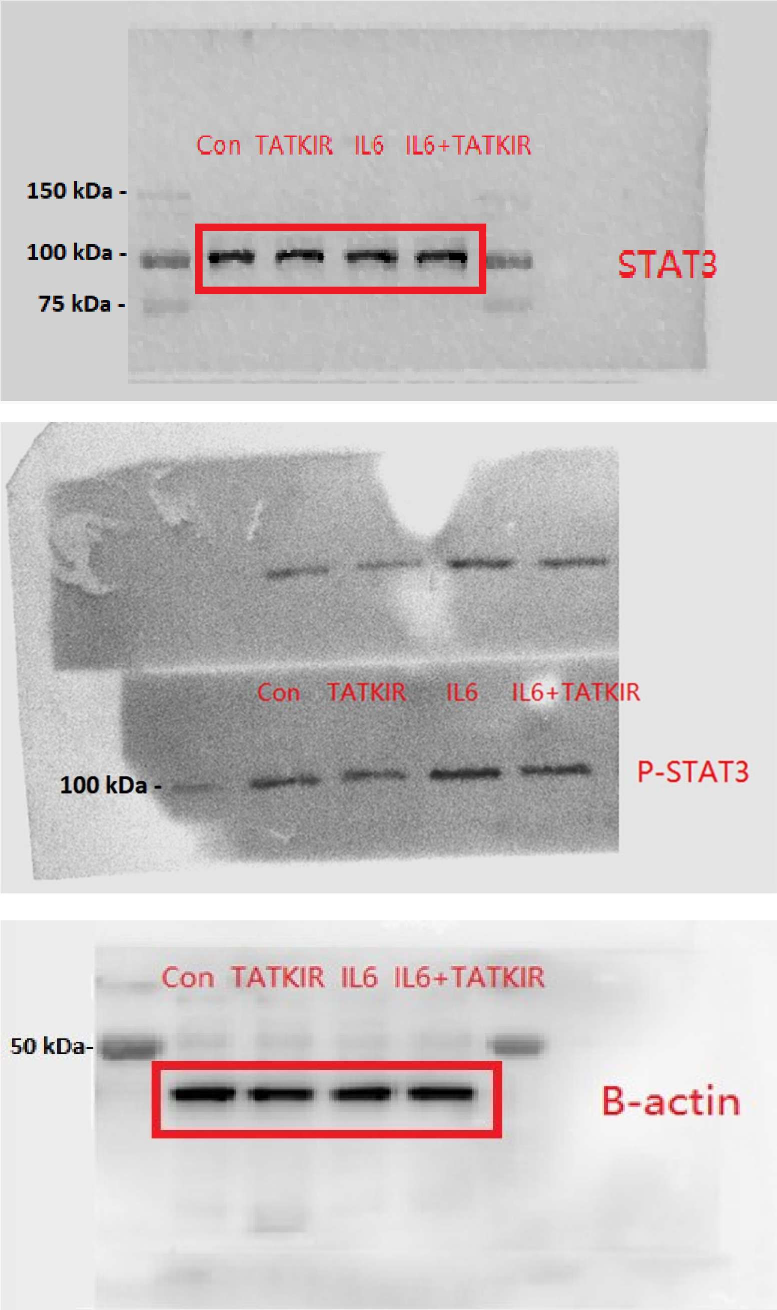

For Figure 4B

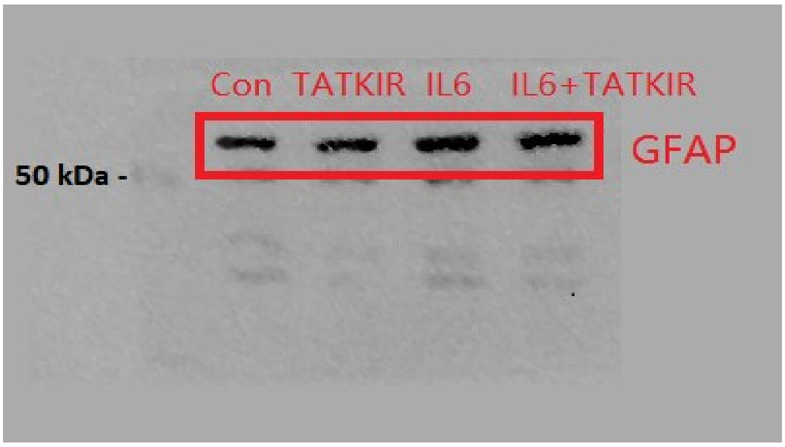

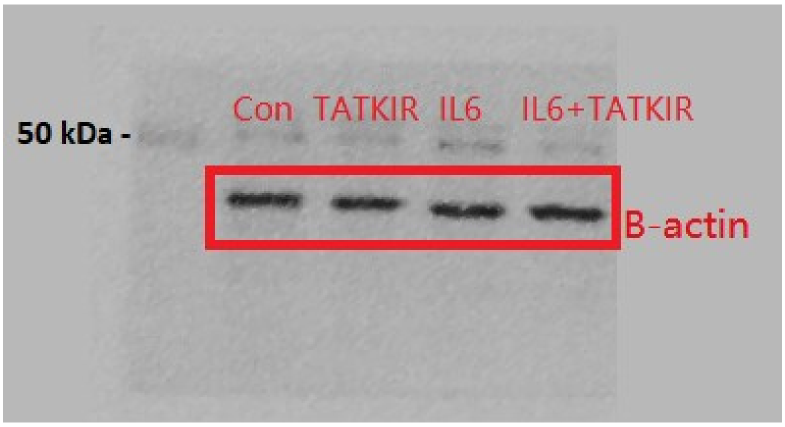

For Figure 4G

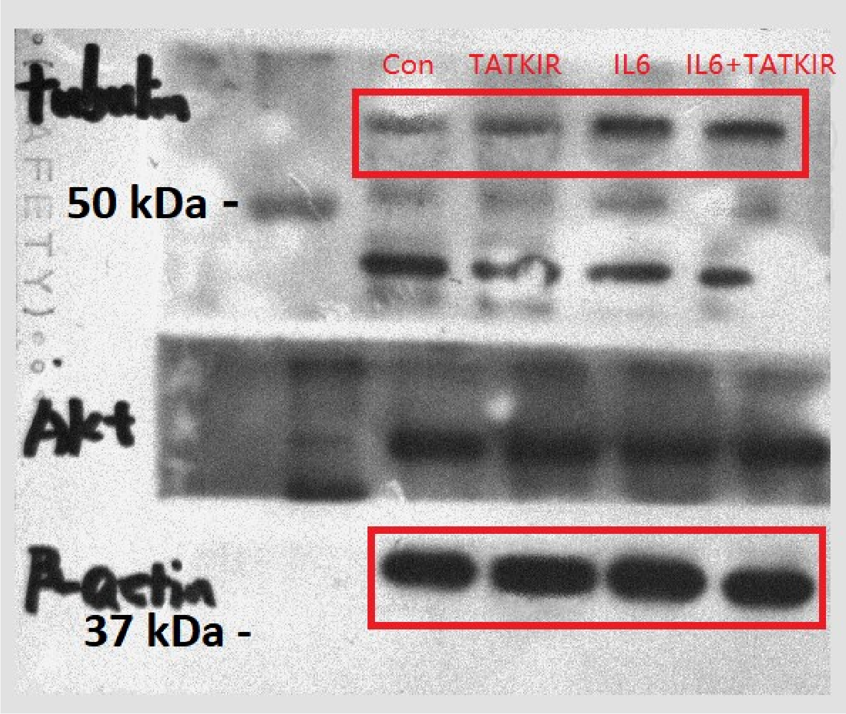

For Figure 4F

Original blot images for Figure 5

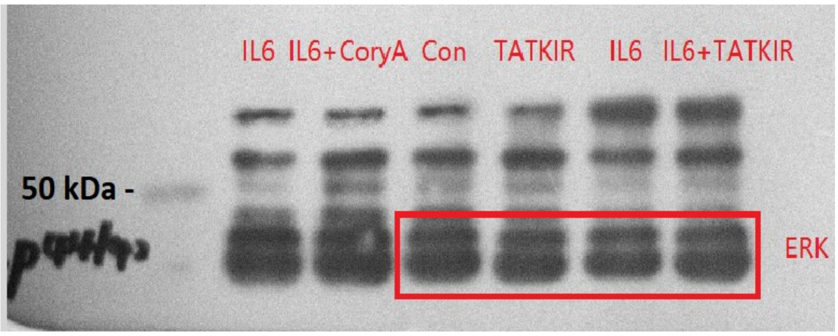

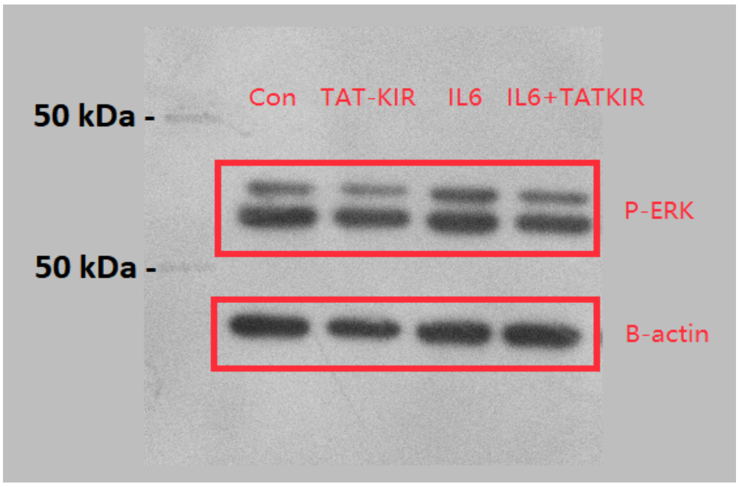

For Figure 5A

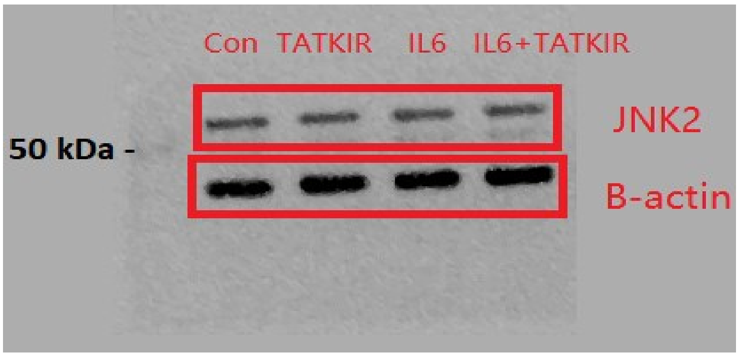

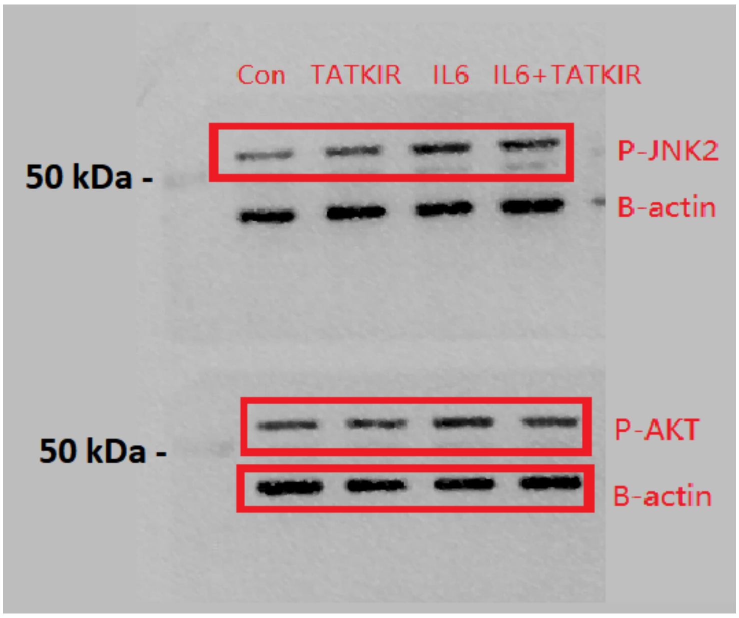

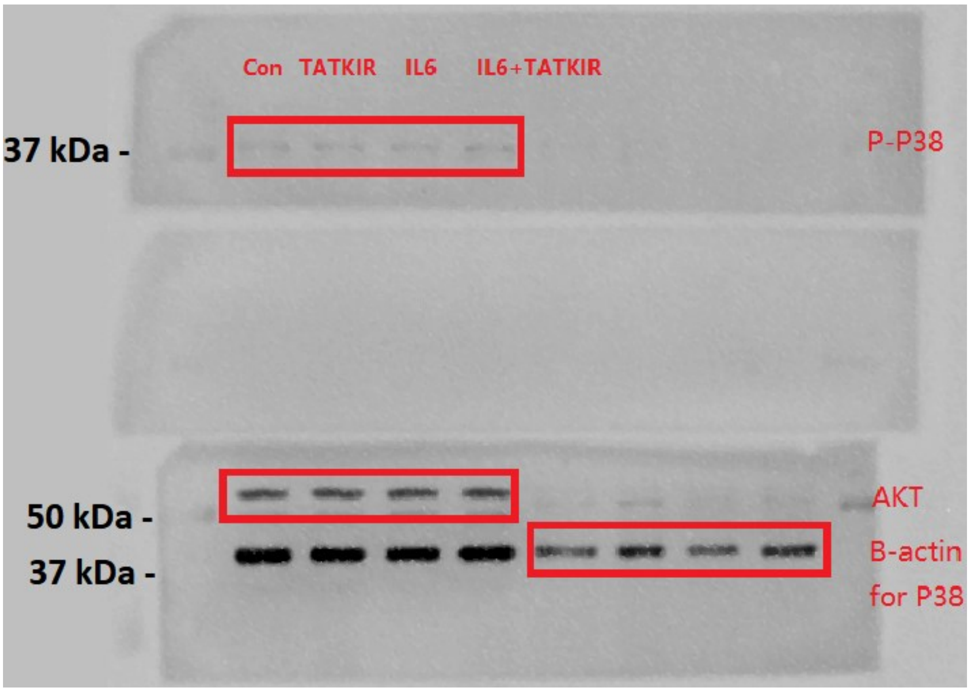

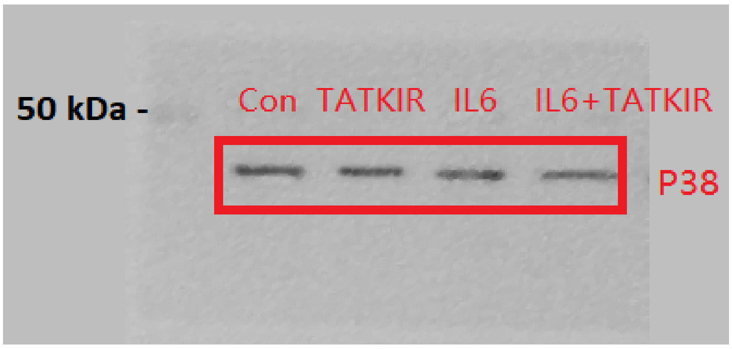

For Figure 5B-D

Original blot images for Figure 6

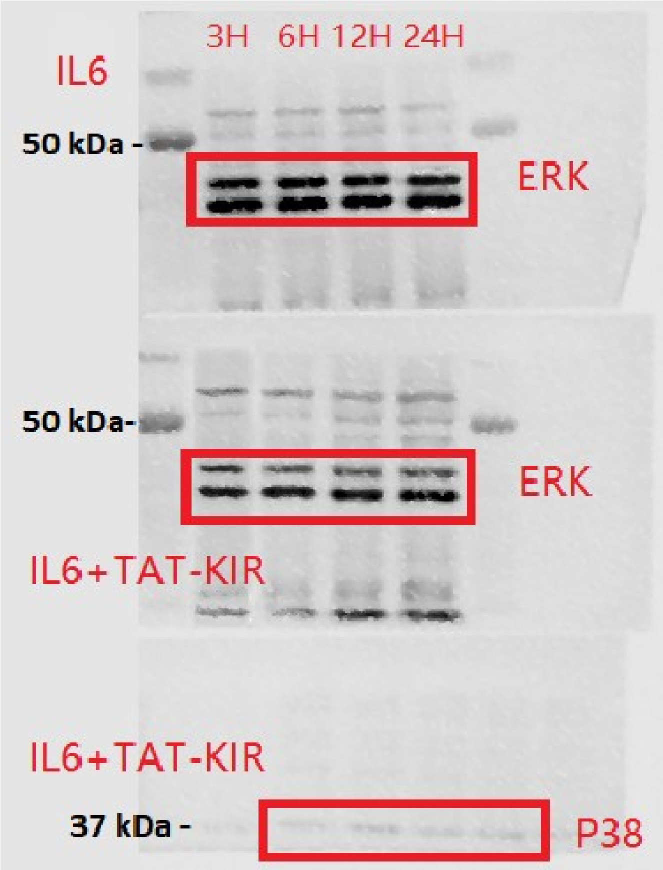

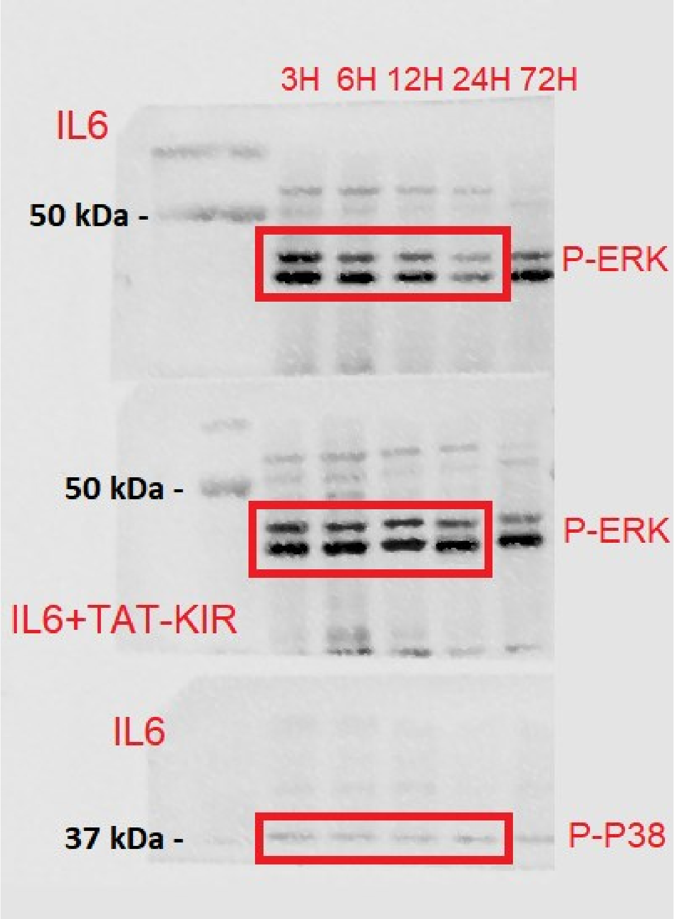

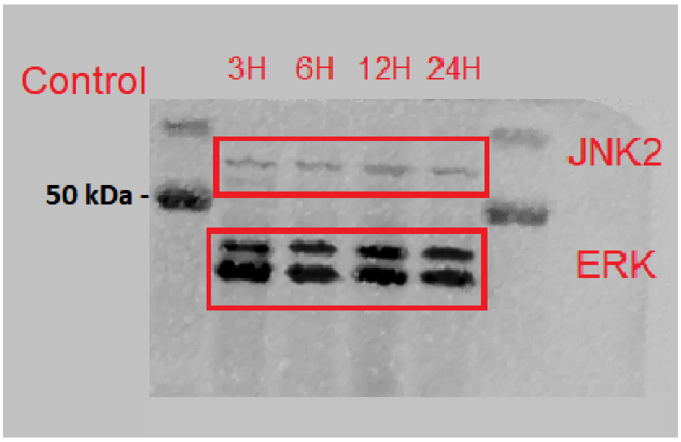
Control
3H 6H 12H 24H
50 kDa -
JNK2
ERK

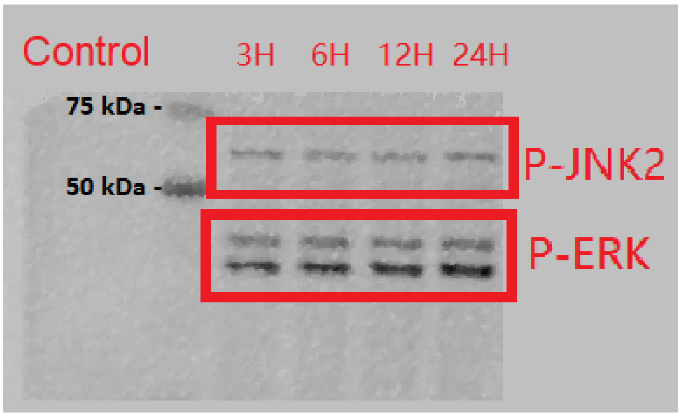
Control
3H 6H 12H 24H
75 kDa -
50 kDa -
P-JNK2
P-ERK

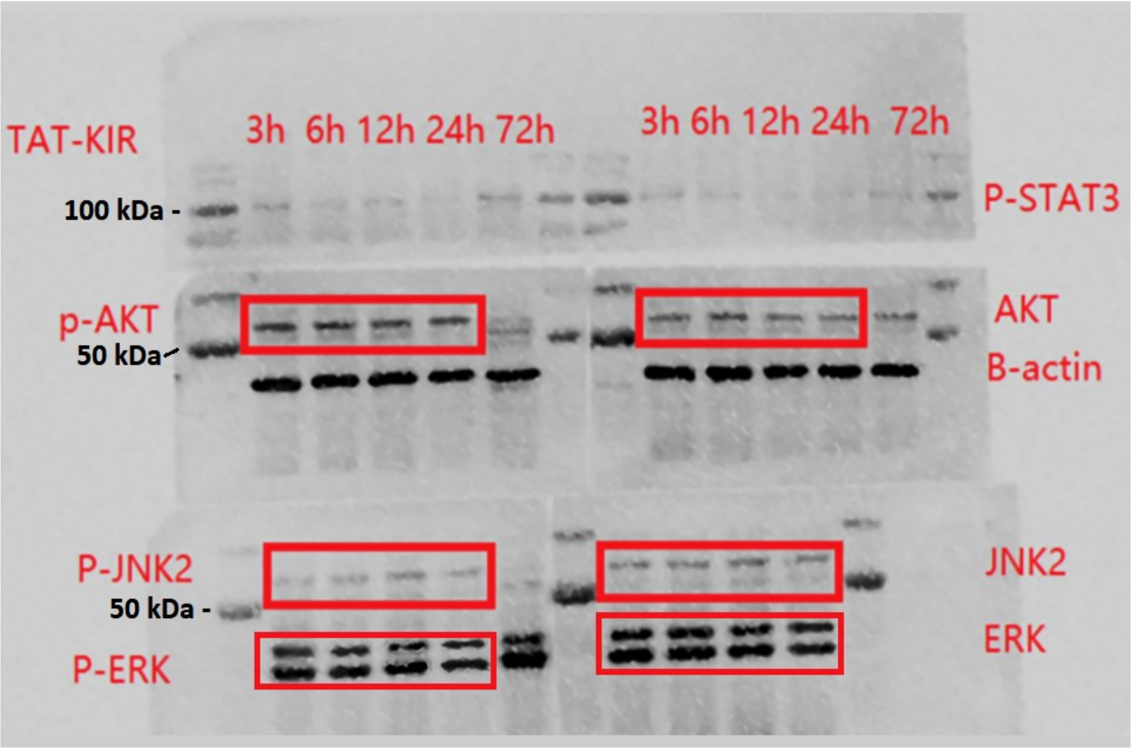
TAT-KIR
3h 6h 12h 24h 72h
3h 6h 12h 24h 72h
100 kDa -
P-STAT3
p-AKT
50 kDa
AKT
B-actin
P-JNK2
50 kDa -
P-ERK
JNK2
ERK

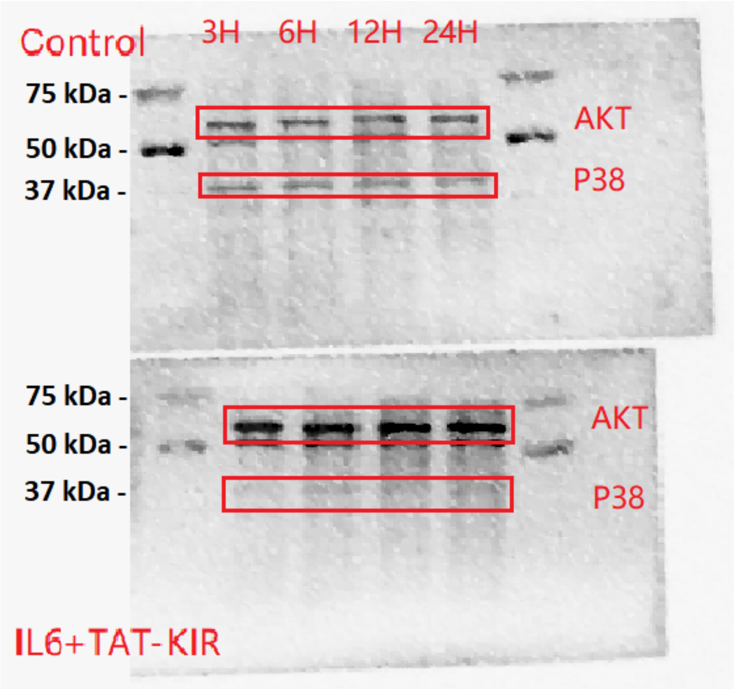
Control
3H
6H
12H
24H
75 kDa -
50 kDa -
37 kDa -
AKT
P38
75 kDa -
50 kDa -
37 kDa -
AKT
P38
IL6+TAT-KIR

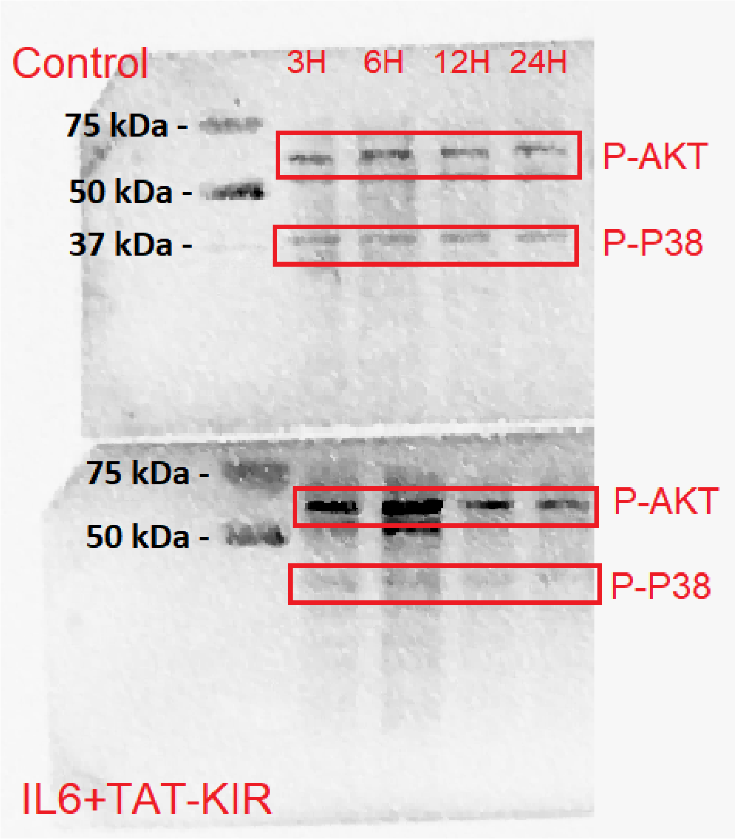
Control
3H
6H
12H
24H
75 kDa -
50 kDa -
37 kDa -
P-AKT
P-P38
75 kDa -
50 kDa -
P-AKT
P-P38
IL6+TAT-KIR

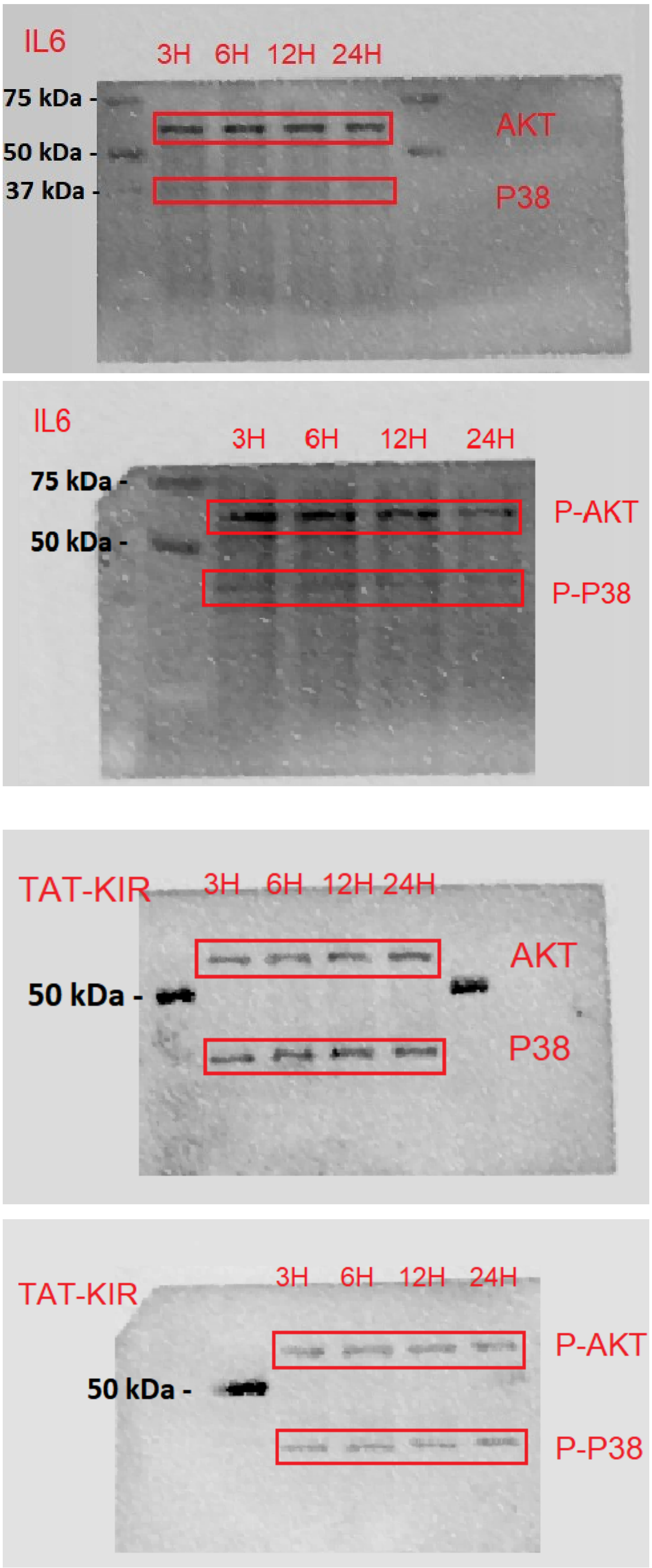
IL6
3H 6H 12H 24H
75 kDa -
50 kDa -
37 kDa -
AKT
P38
IL6
3H 6H 12H 24H
75 kDa -
50 kDa -
P-AKT
P-P38
TAT-KIR
3H 6H 12H 24H
50 kDa -
AKT
P38
TAT-KIR
3H 6H 12H 24H
50 kDa -
P-AKT
P-P38

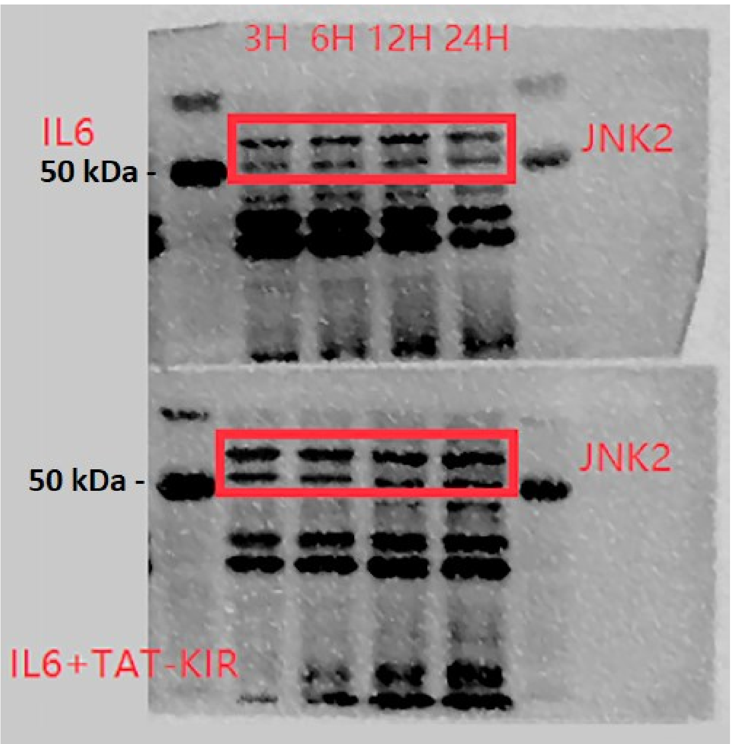
3H 6H 12H 24H
IL6
50 kDa -
JNK2
50 kDa -
JNK2
IL6+TAT-KIR

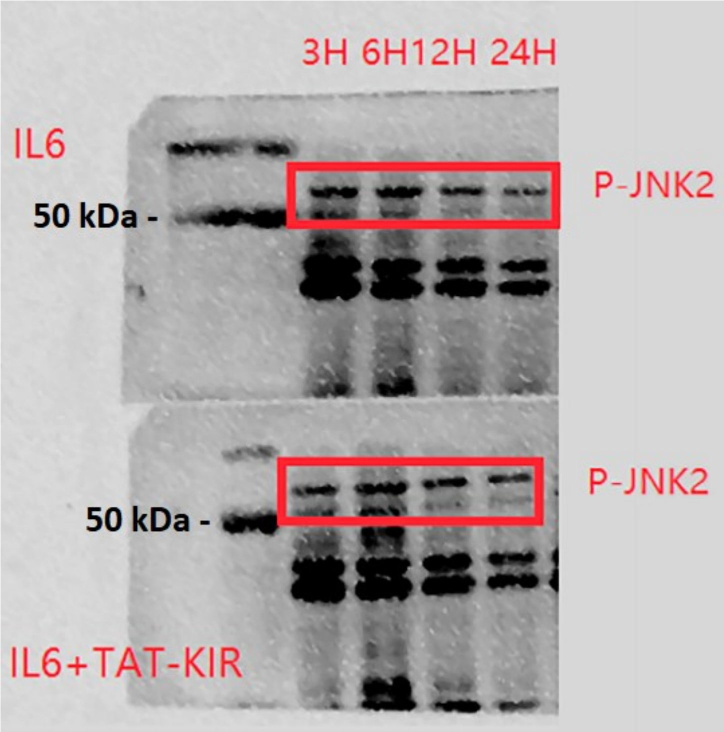
3H 6H12H 24H
IL6
50 kDa -
P-JNK2
50 kDa -
P-JNK2
IL6+TAT-KIR

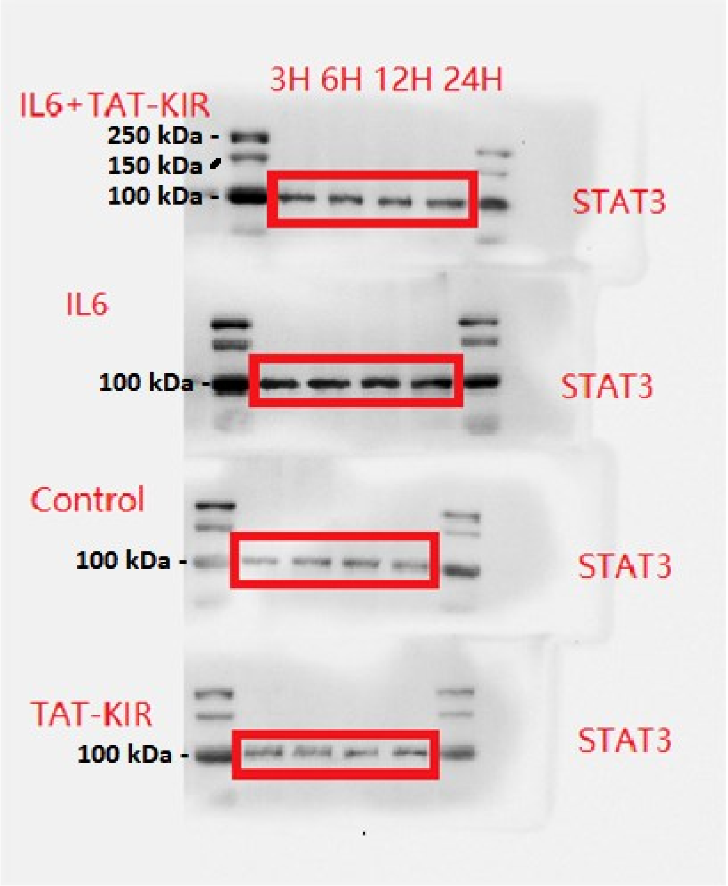
3H 6H 12H 24H
IL6+TAT-KIR
250 kDa -
150 kDa -
100 kDa -
STAT3
IL6
100 kDa -
STAT3
Control
100 kDa -
STAT3
TAT-KIR
100 kDa -
STAT3

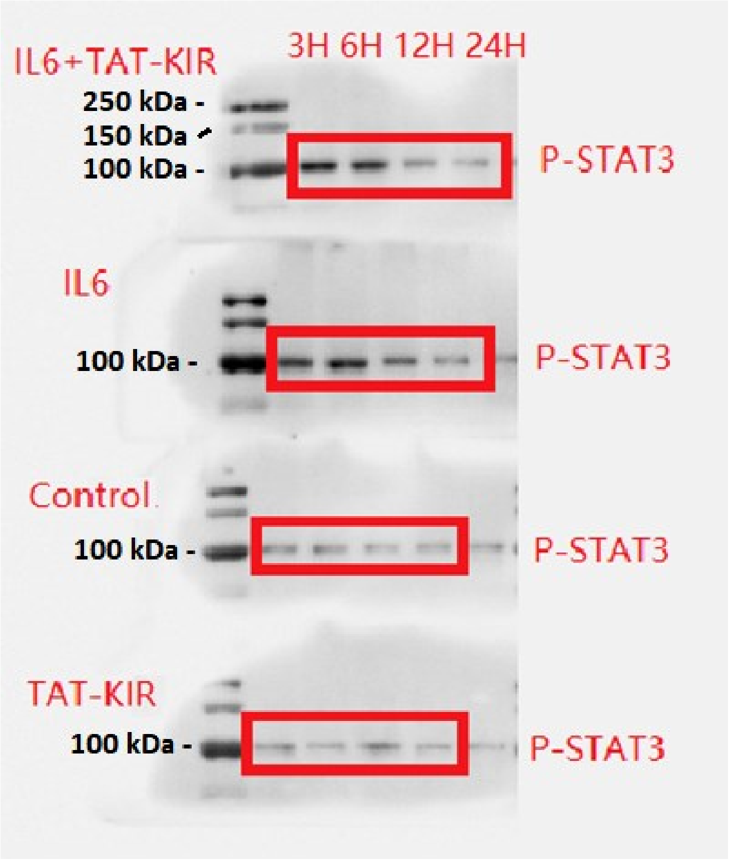
3H 6H 12H 24H
IL6+TAT-KIR
250 kDa -
150 kDa -
100 kDa -
P-STAT3
IL6
100 kDa -
P-STAT3
Control
100 kDa -
P-STAT3
TAT-KIR
100 kDa -
P-STAT3
